# Supplementary material for: Pathway analysis identifies altered mitochondrial metabolism, neurotransmission, structural pathways and complement cascade in retina/RPE/ choroid in chick model of form-deprivation myopia
Source: PeerJ. 2018 Jun 27;6:e5048. doi: 10.7717/peerj.5048 (PMC6026464; doi:10.7717/peerj.5048)
Supplement: Figure S1 — Mean (±SE) measures of anterior chamber and lens thickness during normal development and following induction (FDMI) and recovery (FDMR) from form deprivation. [file peerj-06-5048-s005.pdf]

Supplementary Figure 1

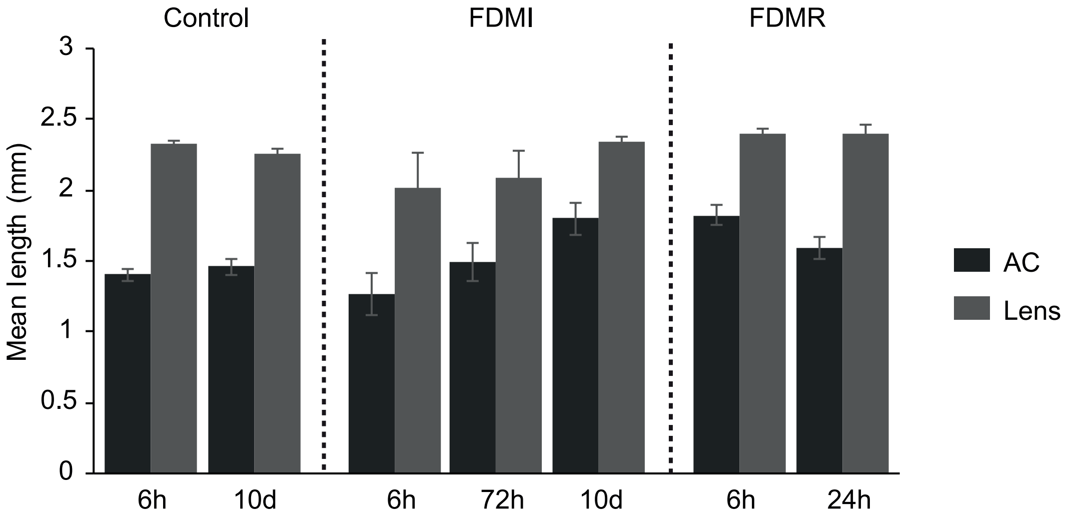

Supplementary Figure 1: Anterior chanber and lens thickness from FDMI and FDMR. Mean (±SE) measures of anterior chamber and lens thickness during normal development and following induction (FDMI) and recovery (FDMR) from form deprivation.
